# Oxidative Stress in Benign Prostatic Hyperplasia: Mechanisms, Clinical Relevance and Therapeutic Perspectives

**DOI:** 10.3390/diseases13020053

**Published:** 2025-02-11

**Authors:** Aris Kaltsas, Timoleon Giannakas, Marios Stavropoulos, Zisis Kratiras, Michael Chrisofos

**Affiliations:** Third Department of Urology, Attikon University Hospital, School of Medicine, National and Kapodistrian University of Athens, 12462 Athens, Greece; ares-kaltsas@hotmail.com (A.K.); tgiannakas@gmail.com (T.G.); stamarios@yahoo.gr (M.S.); zkratiras@gmail.com (Z.K.)

**Keywords:** benign prostatic hyperplasia, oxidative stress, inflammation, reactive oxygen species

## Abstract

Background/Objectives: Benign prostatic hyperplasia (BPH) is among the most common conditions affecting men as they age, resulting in lower urinary tract symptoms (LUTS) that can profoundly impact quality of life. While historically attributed primarily to androgenic imbalances, current evidence implicates additional factors—particularly oxidative stress (OS) and chronic inflammation—in BPH pathogenesis. This review aims to synthesize research on the interplay between OS, inflammation, and hormonal regulation in BPH, emphasizing their clinical relevance and potential therapeutic implications. Methods: A comprehensive review of peer-reviewed literature was conducted focusing on mechanistic studies, clinical trials, and observational reports. Searches included data on ROS generation, antioxidant capacity, inflammatory mediators, and their contribution to pathological prostatic overgrowth. Potential interventions targeting OS—such as antioxidant supplementation, anti-inflammatory drugs, vitamin D receptor agonists, and phytotherapeutics—were also evaluated for their efficacy and safety profiles. Results: Chronic inflammation and OS were consistently identified within hyperplastic prostate tissue. Excessive ROS production, diminished antioxidant defense, and sustained cytokine release create a proproliferative and antiapoptotic environment, accelerating disease progression. Metabolic comorbidities (e.g., obesity, insulin resistance) further exacerbate these imbalances. Standard therapies (α-blockers and 5-ARIs) effectively relieve symptoms but do not directly address the oxidative–inflammatory axis. Emerging evidence suggests that pharmacological and dietary approaches targeting OS and inflammation may reduce prostate volume expansion and alleviate LUTS. Conclusions: Findings indicate that OS and inflammation are key contributors to BPH progression. Incorporating antioxidant and anti-inflammatory strategies alongside conventional treatments holds promise for improving clinical outcomes and patient quality of life. Future research should focus on validating OS-specific biomarkers and optimizing personalized therapy regimens.

## 1. Introduction

Benign prostatic hyperplasia (BPH) is widely acknowledged as a global public health concern, particularly in older men, where its prevalence can exceed 70–90% by the eighth decade of life [1,2]. Although historically conceptualized as a hormone-dependent enlargement primarily regulated by testosterone and its potent metabolite dihydrotestosterone, this traditional paradigm has not fully accounted for the substantial variability in clinical outcomes [3]. Accumulating evidence emphasizes that chronic inflammation and oxidative stress (OS) constitute critical, yet underexplored, facets of BPH pathogenesis [4]. Inflammatory infiltrates—often consisting of macrophages, lymphocytes, and neutrophils—are frequently identified in hyperplastic prostate tissue, and these immune cells secrete cytokines that are capable of perpetuating tissue injury [5]. Concurrently, reactive oxygen species generated through normal metabolic processes or heightened inflammatory activity can overwhelm intrinsic antioxidant defenses, thus promoting fibromuscular proliferation and disrupting apoptotic mechanisms [6]. Although some individuals exhibit histologic signs of BPH yet remain asymptomatic, many others progress to troublesome lower urinary tract symptoms (LUTS), including urinary frequency, nocturia, and obstructive voiding difficulties [7,8].

Despite advancements in understanding the roles of inflammation and OS in prostatic hyperplasia, significant gaps persist. For instance, it remains unclear which patients among those demonstrating early prostatic changes will develop severe LUTS or face complications such as acute urinary retention [9]. Moreover, biomarkers that accurately reflect local inflammatory activity or oxidative damage—such as malondialdehyde or proinflammatory cytokines—have yet to be validated for routine clinical application [10]. Standard therapies, including alpha-blockers (α-blockers) and 5-alpha reductase inhibitors, alleviate symptoms by targeting smooth muscle tension or androgen-driven proliferation but do not directly modulate OS or inflammatory cascades. Emerging interventions—such as nonsteroidal anti-inflammatory drugs, vitamin D receptor agonists, and various antioxidants and phytotherapeutics—offer theoretical benefits by dampening proinflammatory or oxidative pathways; however, robust, large-scale evidence remains limited [11].

Against this backdrop, this manuscript provides a comprehensive appraisal of the interactions among hormones, chronic inflammation, and OS in BPH alongside an examination of potential adjunctive therapies that address these deeper molecular drivers. The analysis may help bridge existing knowledge gaps and inform the development of more comprehensive and potentially personalized approaches to managing this highly prevalent urological disorder.

## 2. Materials and Methods

### 2.1. Literature Search Strategy

A comprehensive literature search was conducted to identify peer-reviewed articles examining the interplay between oxidative stress, inflammation, hormonal regulation, and BPH. Three electronic databases—PubMed, Scopus, and Web of Science—were selected due to their broad coverage of biomedical, life sciences, and multidisciplinary research. The searches encompassed publications from the inception of each database through December 2024, ensuring the inclusion of both foundational studies and the most recent investigations.

### 2.2. Search Terms and Eligibility Criteria

Relevant Medical Subject Headings (MeSHs) and keywords were employed either individually or in combination with Boolean operators (“AND”, “OR”). These included “oxidative stress”, “inflammation”, “benign prostatic hyperplasia”, “BPH”, “prostate”, “reactive oxygen species”, “apoptosis”, “proliferation”, “hormonal regulation”, “5-alpha reductase inhibitors”, “alpha-blockers”, “prostatic inflammation”, and “antioxidant therapy”. Studies were considered eligible if they (i) provided data or analyses on the contribution of oxidative stress or inflammation to BPH pathogenesis, progression, or management and (ii) included mechanistic, clinical, or therapeutic insights relevant to BPH. Both original research articles (e.g., clinical trials, in vitro or in vivo experiments, observational studies) and review articles were included for a comprehensive narrative. Publications not written in English, conference abstracts lacking methodological details, and articles focusing solely on non-urological conditions were excluded.

### 2.3. Study Selection and Data Extraction

All retrieved citations were imported into reference management software (EndNote) to facilitate the identification and removal of duplicates. Subsequently, titles and abstracts were screened according to the predefined inclusion and exclusion criteria. Full-text articles were then evaluated to confirm their relevance. Key findings were extracted and categorized under the following thematic areas: (i) oxidative stress mechanisms in BPH, (ii) inflammation-mediated pathways contributing to prostatic hyperplasia, (iii) hormonal interplay in BPH progression, (iv) diagnostic or prognostic biomarkers of oxidative stress and inflammation, (v) standard and emerging treatments, and (vi) existing gaps and future directions.

### 2.4. Quality Assessment

An informal appraisal of methodological quality was performed for each included study. This assessment involved examining the clarity of the study’s objectives, the comprehensiveness of experimental or clinical methods, the appropriateness of outcome measures, and the robustness of the reported data related to oxidative stress or inflammatory markers in BPH. Studies lacking sufficient methodological detail or clarity in reporting key parameters were excluded.

### 2.5. Data Synthesis

Relevant information from eligible articles was synthesized into sections reflecting the multifaceted nature of BPH. Because the goal was to provide a broad, integrative view of the oxidative–inflammatory axis rather than a quantitative aggregation, data were combined in a narrative framework. Particular attention was directed toward how oxidative stress and inflammation intersect with hormonal dysregulation to influence disease initiation, progression, and clinical manifestation. Contradictory findings and unresolved questions were highlighted to emphasize areas requiring further research. The final structure of this review was designed to illuminate the molecular underpinnings of BPH while also providing insight into potential therapeutic targets and the necessity for more personalized management strategies.

## 3. Epidemiology, Anatomy and Inflammatory–Oxidative Mechanisms in BPH

### 3.1. Global Epidemiology and Clinical Symptomatology

BPH constitutes a considerable public health issue worldwide due to its economic impact and the substantial burden it places on the quality of life among aging men [12].

Epidemiological data indicate that BPH prevalence rises with increasing age, affecting up to 80% of men by the time they are 70 years old [13,14]. Indeed, the majority of older men eventually develop some histological characteristics consistent with BPH during their lifetimes [15].

This epidemiologic heterogeneity can be partly explained by differences in genetic predisposition, dietary patterns, physical activity levels, and comorbid conditions (e.g., metabolic syndrome) [16]. Notably, prostate enlargement does not necessarily result in clinical LUTS for every individual. The presence and severity of LUTS depend on factors such as prostate volume, bladder responsiveness, inflammatory changes, and the degree of prostatic obstruction [17]. Clinically, BPH can manifest with obstructive symptoms (e.g., hesitancy, straining, intermittent flow, weak stream, incomplete emptying) or irritative symptoms (e.g., frequency, urgency, nocturia). The International Prostate Symptom Score (IPSS) remains the standard assessment tool, allowing clinicians to classify symptom severity—from mild to severe—and to gauge the impact on daily activities and psychosocial well-being [18].

### 3.2. Prostatic Zonal Anatomy and Cellular Interplay

The prostate is anatomically arranged as a conical organ under the bladder encircling the urethra. Classical zonal anatomy partitions it into peripheral, transition, central, as well as the anterior fibromuscular zone [19]. BPH largely arises within the transition zone and periurethral region, pushing on the urethral lumen and triggering the mechanical obstruction that underlies many voiding symptoms. Histologically, the prostate contains a glandular epithelium that produces prostatic fluid and a stromal compartment composed of fibroblasts, smooth muscle cells, and an extracellular matrix. These smooth muscle elements are partially responsible for regulating prostatic tone and can augment obstruction when they contract. Both stromal and epithelial components contribute to the emerging nodular expansions observed on imaging and in histopathological specimens [20]. Although the androgen receptor is abundantly expressed throughout prostatic tissue, androgen levels alone do not fully account for why BPH develops in some individuals but not others. As described below, other subtler processes, such as oxidative stress-induced hyperplasia, play a pivotal role [21].

### 3.3. Chronic Inflammation as a Contributor to BPH Pathogenesis

Histological analyses of resected BPH tissue frequently reveal moderate to marked inflammatory cell infiltrates, suggesting that inflammation is an active driver of aberrant prostatic growth rather than an incidental finding [22]. The infiltrates typically consist of T-lymphocytes and macrophages alongside occasional plasma cells and neutrophils [23]. Chronic inflammation may arise from multiple overlapping sources, including subclinical bacterial infections in prostatic ducts, urinary reflux of chemical irritants, mechanical microtrauma over many years, or autoimmune responses [10]. Moreover, metabolic syndrome, insulin resistance, and obesity can aggravate these localized prostatic inflammatory processes, reinforcing a cycle of tissue injury [24].

Once inflammation is established, proinflammatory cytokines such as interleukin-6 (IL-6), tumor necrosis factor-alpha (TNF-α), and interleukin-8 (IL-8) activate signaling cascades that drive cellular proliferation and impede normal apoptotic turnover, fostering nodular hyperplasia [25]. Consequently, chronic inflammation and nodular hyperplasia often coexist in the transition zone of the prostate, with cytokine-mediated remodeling being implicated in disease progression [26]. Clinically, pronounced inflammatory activity correlates with a heightened risk of disease progression, acute urinary retention, and more severe LUTS [27].

### 3.4. Emerging Perspectives on Oxidative Stress in BPH

OS, a condition defined by excessive ROS relative to antioxidant defense systems, is increasingly regarded as a critical factor in BPH. ROS include superoxide anion, hydrogen peroxide, hydroxyl radicals, and related molecular species generated endogenously through mitochondrial oxidative phosphorylation, inflammation, or enzymatic activities, such as those mediated by xanthine oxidase. Excessive ROS production can accelerate damage to lipids, proteins and nucleic acids while also altering key signaling pathways that control cell survival proliferation and inflammation [28]. Chronic inflammation intensifies local ROS levels as immune cells release reactive oxygen and nitrogen intermediates in their attempt to neutralize pathogens or remove damaged tissues. Alongside this, men with aging prostates often exhibit diminishing activity of vital antioxidant enzymes, including superoxide dismutase, glutathione peroxidase and catalase. These enzymes normally detoxify superoxide or hydrogen peroxide, limiting the damage to cellular structures. The synergy among decreased antioxidant capacity, excess ROS, and chronic prostatic inflammation creates a vicious cycle that perpetuates hyperplastic changes. Mitochondrial dysfunction may also contribute to prostatic OS because, as the prostate ages, the electron transport chain can become leaky, thereby increasing superoxide production and undermining ATP production. Another concern is that OS can degrade critical DNA repair mechanisms, leading to genomic instability. This risk theoretically extends to malignant transformation, although the specific sequence bridging BPH and prostate carcinoma remains an area of active investigation [29].

## 4. Oxidative Stress Pathways in BPH

### 4.1. Defining Oxidative Stress and Its Primary Sources

OS is a biological condition that occurs when free radicals, particularly ROS, exceed the neutralizing capacity of antioxidant defenses. This typically manifests in tissues with high metabolic activity or chronic inflammatory stimuli. Mitochondria produce ROS as a normal byproduct of electron transport for generating ATP. Under healthy conditions, antioxidant networks detoxify these radicals, maintaining a redox equilibrium that supports physiological signaling. However, factors such as inflammation, environmental toxins, radiation, poor nutritional states, metabolic syndrome, and advanced age tip this balance toward the overproduction of ROS. This environment fosters ongoing biomolecular damage, which, in turn, drives tissue proliferation [30].

### 4.2. Intracellular Signaling Roles of ROS

Although OS often carries a negative connotation, ROS can function as essential mediators of normal cell signaling. At regulated concentrations, moderate amounts of hydrogen peroxide or superoxide act as second messengers that modulate kinases, phosphatases, and transcription factors—such as mitogen-activated protein kinases (MAPKs), nuclear factor kappa B (NF-κB), and activator protein 1 (AP-1). Through these mechanisms, ROS influence cell proliferation, differentiation, senescence, and apoptosis [31].

In the aging prostate, especially in the presence of chronic low-level inflammation, ROS may remain persistently elevated, driving proinflammatory gene expression, fostering abnormal proliferation, and hampering normal apoptotic controls. The resultant net increase in cellular mass within the transition zone leads to the characteristic nodules observed in BPH. Given ROS’s dualistic nature, complete elimination of all radicals might be detrimental. Instead, the goal is to fine-tune their levels and restore physiological redox signaling [32].

### 4.3. Antioxidant Deficiencies and Their Impact on BPH

A key finding in numerous BPH studies is the reduced activity of critical antioxidant enzymes in prostatic tissue. Superoxide dismutase (SOD) converts superoxide into hydrogen peroxide, glutathione peroxidase detoxifies hydrogen peroxide, and catalase further breaks down hydrogen peroxide into water and oxygen. Low levels or reduced activity in these enzymes compromise the effective elimination of ROS [33].

Additionally, decreased glutathione—a universal cellular antioxidant—has been reported in hyperplastic prostate tissues and in the serum of men with BPH. Glutathione not only directly scavenges free radicals but also serves as a cofactor for various redox-regulating enzymes. Collectively, these antioxidant deficits enhance OS, perpetuating tissue remodeling, fibroblast infiltration, stromal transformation, and epithelial hyperplasia, all of which are key features of BPH pathogenesis [34].

**Figure 1:**
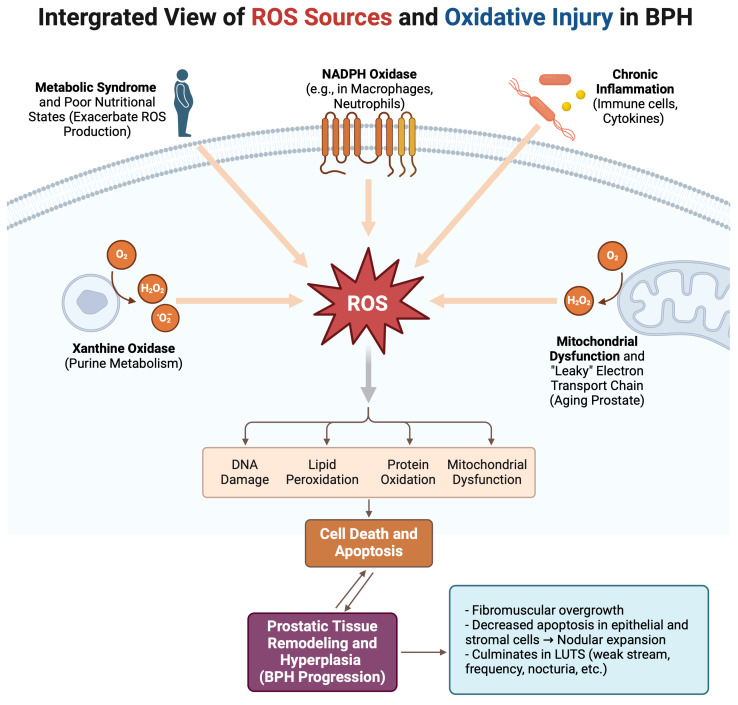
Schematic of ROS generation and oxidative pathways leading to BPH progression. *Created in BioRender. Kaltsas, A. (2025)*, https://BioRender.com/e65o135 (accessed on 13 January 2025).

A visual summary of these oxidative stress pathways and their role in BPH progression is shown in Figure 1 below.

## 5. The Interplay of Apoptosis and Oxidative Stress in BPH Progression

### 5.1. Regulation of Apoptosis in Prostate Homeostasis

A healthy prostate relies on a delicate balance of cell proliferation, regulated by androgens and cell death, mediated by carefully controlled apoptotic pathways. Within normal limits, androgens do not only drive proliferation but also indirectly help maintain adequate apoptotic control. When these processes remain balanced, the prostate’s size and histological architecture remain stable [35,36]. However, in BPH, proapoptotic signals can become relatively reduced, while proproliferative or pro-survival inputs intensify. Chronic OS and inflammation damage or inactivate certain mediators of apoptosis, including tumor protein p53 and certain Bcl-2 family members, thereby favoring cell survival over cell death [37,38]. Moreover, the normal age-related increase in prostate volume has been linked to heightened proliferation rates and diminished apoptosis [39]. Together, these processes create an environment in which accumulating cells are less likely to undergo programmed death, ultimately promoting prostatic overgrowth that is characteristic of BPH.

### 5.2. OS-Mediated Pathways of Cell Death and Survival

OS can trigger several apoptotic or necrotic pathways depending on the intensity and duration of the insult. High acute concentrations of ROS typically initiate DNA and membrane damage that leads to activation of stress kinases, including c-Jun N-terminal kinase (JNK) or p38 mitogen-activated protein kinase (p38 MAPK) [40,41]. These stress kinases can prompt intrinsic apoptosis (by causing mitochondrial membrane permeabilization and cytochrome-c release) or extrinsic apoptosis (by upregulating death receptors) [41,42]. However, in chronic prostatic inflammation and mild but sustained OS conditions, the situation becomes more complex. Sublethal levels of ROS continuously strike cells, leading them to develop adaptive pro-survival responses that paradoxically reduce apoptosis. Simultaneously, the environment fosters low-grade DNA damage, which can produce senescent phenotypes or hyperplastic transformations. Molecular regulators, such as the preapoptotic protein p66Shc, which modulate redox states can also alter the apoptotic threshold. This phenomenon suggests that repeated moderate oxidative insults over time in BPH may reduce apoptosis more than if the stress were acute and overwhelming, thereby facilitating abnormal cell accumulation rather than programmed cell death [43,44].

### 5.3. Synergistic Interactions Between Apoptosis and Inflammation

OS rarely acts alone in prostatic pathology. Instead, it closely interacts with inflammatory signals in a positive feedback loop. Proinflammatory cytokines like IL-6 or TNF-a promote the generation of more radicals through various mechanisms, including the upregulation of NADPH oxidases and respiratory bursts in immune cells [45]. Elevated ROS levels, in turn, activate nuclear factor kappa B (NFK-b), which induces further cytokine and chemokine production, recruiting more immune cells. This cycle disrupts normal proapoptotic cascades. The net effect in BPH is that inflamed prostatic tissue experiences reduced apoptosis and greater proliferation [46,47].

## 6. Hormonal Interplay and Oxidative Stress in BPH

### 6.1. Androgenic Regulation and ROS Generation

Androgens, primarily testosterone and dihydrotestosterone, are crucial for prostatic maintenance and secretory activity [48]. When androgens bind androgen receptors in epithelial and stromal cells, gene transcription that favors cell growth is initiated [49]. At the same time, upregulated metabolic demands can escalate mitochondrial ROS production [50]. Under healthy or younger conditions, antioxidant mechanisms buffer this surge. With advanced age, these protective enzymes diminish, resulting in heightened OS [51,52]. If additional factors, like inflammation or suboptimal diet, come into play, they can further tilt the prostatic microenvironment even more toward hyperproliferation [49]. Some data suggest that chronic low-grade androgenic stimuli, combined with cytokine-driven inflammation, are particularly conducive to creating an environment that fosters hyperplasia [49]. Thus, while androgens remain a central actor in prostatic physiology, they interact with the entire redox and immunological framework to influence the pace of BPH development.

### 6.2. Estrogenic Influencens and Prostate Pathobiology

Estrogens have historically been overshadowed by androgens in prostate research; however, studies demonstrate that both estrogen receptor alpha (ERα) and estrogen receptor beta (ERβ) are expressed in prostatic tissues at varying densities [53,54]. Experimental rodent models demonstrate that chronic exposure to elevated estradiol levels can provoke inflammatory infiltration, OS signatures, and proliferative nodules reminiscent of BPH [55]. One proposed mechanism is that estrogens can alter cytokine output and antioxidant enzyme expression, thereby driving the tissue toward chronic low-grade damage and growth [56]. Another possibility is that estrogens may intersect with androgen receptor pathways, modulating or amplifying the effect of dihydrotestosterone or testosterone [53]. This interplay appears to be context-dependent, with the balance of ERα and ERβ expression dictating whether the net result is proliferative [57]. Unraveling how estrogens influence BPH has led to suggestions that partial inhibition of certain estrogenic pathways might help mitigate disease progression [58]. Nonetheless, this remains a relatively underexplored area compared to standard hormone blocking therapies that directly target androgens.

### 6.3. Metabolic Syndrome and Hormonal–Inflammatory Interactions

Metabolic syndrome, characterized by central obesity, hypertension, dyslipidemia, and hyperglycemia, has been strongly linked to a range of inflammatory disorders [59]. It also alters sex hormone profiles, often decreasing total testosterone while increasing circulating free estrogen through heightened aromatase activity in adipose tissue. This hormonal imbalance can exacerbate or predispose individuals to BPH [48]. Additionally, metabolic syndrome triggers systemic OS. Excess glucose promotes the formation of advanced glycation end products (AGEs) through the non-enzymatic attachment of glucose to proteins, lipids, or nucleic acids [60]. Beyond eliciting an inflammatory response by binding to the receptor for advanced glycation end products (RAGEs), these AGE-RAGE interactions also enhance the production of ROS, further exacerbating OS [52]. Meanwhile, adipose tissue secretes substantial amounts of TNF-α and IL-6, which not only infiltrate the prostate but also exacerbate mitochondrial dysfunction and fuel local ROS production [49]. Men with metabolic syndrome often present with more severe LUTS or advanced hyperplasia. Interventions such as weight reduction, improved insulin sensitivity, and anti-inflammatory diets have been associated with reduced LUTS severity in observational studies [56], suggesting that addressing metabolic risk factors may indirectly stabilize or slow BPH progression.

Importantly, metabolic syndrome can also facilitate local arteriosclerosis and ischemia within the lower urinary tract, including the prostate. Several studies have demonstrated that ischemia resulting from atherosclerotic changes in prostatic arteries fuels oxidative stress and inflammation—both hallmarks of BPH pathogenesis [61,62]. In human surgical specimens, for example, severe local atherosclerosis correlates with larger prostate volumes and upregulated expression of malondialdehyde (MDA), hypoxia-inducible factor (HIF)-1α, and key profibrotic mediators such as transforming growth factor (TGF)-β1 and basic fibroblast growth factor (bFGF) [61]. Chronic hypoxia in ischemic prostate tissue appears to be the critical trigger of these oxidative pathways, ultimately contributing to fibromuscular proliferation. Likewise, in rodent models, restoring blood flow to the prostate (e.g., by administering nicorandil) reduces oxidative stress markers and ameliorates hyperplastic changes [63]. Collectively, these findings strongly suggest that managing vascular health—particularly atherosclerosis—could be a valuable strategy in preventing or slowing BPH progression by mitigating ischemia-driven oxidative injury.

## 7. Inflammation, Oxidative Stress, and Clinical Implications in BPH

### 7.1. Chronic Inflammation as a Catalyst for Prostatic Overgrowth

Multiple lines of evidence implicate chronic prostatic inflammation as a pivotal driver of BPH. Histological assessments frequently reveal hyperplastic nodules coexisting with inflammatory infiltrates, suggesting that persistent inflammation underlies disease progression [64,65]. Such inflammation may originate from bacterial infiltration, including subclinical infections [66], autoimmune-like responses, or repeated mechanical stress and microtrauma [65]. In each scenario, ongoing cytokine release and oxidative bursts disrupt normal cell turnover. This mismanaged tissue repair fosters gradual tissue overgrowth [45,67]. Clinically, heightened inflammatory activity often correlates with more severe symptomatology—for instance, acute urinary retention or a rapidly escalating IPSS [68]. Chronic inflammation also primes the local immune environment for cross-talk with OS, further amplifying fibromuscular proliferation and hyperplastic nodule formation [45].

### 7.2. Inflammatory Cell Dynamics and Reactive Oxygen Species

Neutrophils, macrophages, and even T-lymphocytes release ROS as part of their innate defense mechanisms [69,70]. In the prostates of men with BPH, these immune cells accumulate in significant numbers. They unleash superoxide anion, nitric oxide, and hydrogen peroxide, which directly damage epithelial and stromal cells [69]. Damaged or dying cells, in turn, release signals that recruit additional immune cells, perpetuating this inflammatory cycle [64,71]. Concurrently, cyclooxygenase-2 (COX-2) upregulation in inflamed tissue elevates prostaglandin levels, fueling vascular changes and further proinflammatory signaling [45]. Prolonged oxidative damage to key cellular structures—particularly in stromal myofibroblasts and epithelial cells—can alter cell phenotypes, shifting them toward proliferative or fibrotic states [65,67]. Consequently, a chronically inflamed and oxidatively stressed microenvironment emerges, facilitating the progressive development of BPH.

### 7.3. Self-Perpetuating Cycles: The Inflammatory–Oxidative Pathway

Once initiated, the inflammatory–oxidative cycle can become self-sustaining. For example, ROS can activate the nuclear factor kappa B (NF-κB) pathway, which drives the transcription of proinflammatory cytokines such as tumor necrosis factor alpha (TNF-α) and interleukin-6 (IL-6) [65,72]. These cytokines then promote additional immune cell recruitment, intensify local ROS generation, and enhance fibroblast or epithelial proliferation. BPH tissues frequently exhibit elevated NF-κB activity, paralleling the observed pattern of ongoing proinflammatory gene expression [73,74,75]. Standard medical therapies—targeting prostatic smooth muscle tone or androgen levels—often prove insufficient in regard to breaking this cycle. Consequently, earlier interventions that reduce NF-κB activation or mitigate ROS generation are gaining attention as potential therapeutic avenues [75,76,77].

### 7.4. Relationship with Symptom Severity

Clinical trials, such as the Medical Therapy of Prostatic Symptoms (MTOPS) trial, underscore the link between prostatic inflammation and symptomatic progression [78]. In that trial, men who displayed histological prostate inflammation experienced a higher likelihood of BPH complications, including acute urinary retention or a need for surgical intervention [79]. A further analysis reveals that heightened OS markers often coincide with advanced LUTS. For instance, increased malondialdehyde levels—an indicator of OS and lipid peroxidation—can disrupt smooth muscle integrity and compromise urethral patency [80]. Persistent oxidative and inflammatory insults may also irritate local nerve endings, accentuating irritative symptoms such as urgency or nocturia [81,82]. Notably, therapies with anti-inflammatory or antioxidant properties show promise in alleviating symptom burden [77,83]. However, more rigorous investigations are necessary to establish standardized protocols, given the heterogeneity of BPH. Some patients appear to be particularly vulnerable to inflammatory exacerbations and oxidative damage, suggesting a potential role for personalized therapeutic strategies in the future [73].

### 7.5. Potential Biomarkers for Prostatic Inflammation and OS

While C-reactive protein (CRP) remains a common measure of systemic inflammation, it lacks specificity in correlating with the severity or progression of BPH [84]. Consequently, research has expanded to incorporate lipid peroxidation markers, like malondialdehyde, and DNA damage indicators, such as 8-hydroxydeoxyguanosine (8-OHdG) [80]. Preliminary findings indicate that elevated levels of these oxidative biomarkers may predict a higher risk of BPH progression [85,86]. Additionally, proinflammatory mediators (e.g., IL-8) in prostatic fluid or tissue and the neutrophil–lymphocyte ratio in blood are emerging as potential indicators of both local and systemic inflammatory states [85,87]. Before these biomarkers can be integrated into routine practice, however, they necessitate robust prospective validation. Moreover, a composite biomarker panel may offer superior prognostic accuracy compared to any single marker, potentially guiding early intervention and treatment monitoring in patients at elevated risk [71,81].

### 7.6. Oxidative Stress and Differentiating BPH from Prostate Cancer

Differentiating BPH from prostate cancer is a clinical challenge, as both conditions can elevate serum prostate-specific antigen (PSA) and often coexist in older populations [88,89]. Chronic inflammation and OS contribute to each pathology, although in distinct ways. For example, proliferative inflammatory atrophy (PIA), characterized by atrophy and increased proliferation, may arise from persistent inflammation and exhibit heightened OS markers alongside diminished antioxidant defenses [90]. PIA lesions have been hypothesized to precede malignant transformation when genomic instability accumulates [90]. Nevertheless, most men with BPH do not progress to cancer, revealing only partial overlap between these conditions [91,92]. Understanding how OS differentially affects benign and malignant prostatic growth is therefore paramount. Although BPH remains a non-malignant disorder, it shares certain microenvironmental elements with early oncogenesis [65,90]. Hence, comprehensive diagnostic approaches—incorporating traditional assessments, novel biomarkers, and imaging—should be employed, guided by an appreciation for the complex roles of inflammation and OS in both benign and malignant processes [75,93].

## 8. Therapeutic and Preventive Strategies Targeting Oxidative Stress in BPH

### 8.1. Conventional Pharmacological Treatments

The cornerstone treatments for BPH revolve around α-blockers, such as tamsulosin, alfuzosin and silodosin, and 5-ARIs (5-alpha reductase inhibitors), such as finasteride and dutasteride. A-blockers relax prostatic muscle and the bladder neck smooth muscle, improving urinary flow, while 5-ARIs reduce the androgenic stimulus that drives prostate enlargement [94,95,96]. Combination therapy has demonstrated synergistic benefits, significantly lowering the risk of acute urinary retention and disease progression in men with larger prostate volumes and moderate-to-severe LUTS [96].

Although α-blockers do not directly function as antioxidants, several studies suggest that they can indirectly lower oxidative stress within the prostate by enhancing local blood flow to lower urinary tract organs. Increased perfusion not only helps alleviate ischemia-induced oxidative injury but may also suppress prostate cell proliferation [97,98]. Animal models, for instance, have shown that improved prostatic blood flow under α-blockade correlates with reduced levels of oxidative markers and attenuated tissue remodeling [99].

However, despite their efficacy in providing symptomatic relief, neither α-blockers nor 5-ARIs directly target the OS-inflammation cycle [65]. As a result, patients who remain symptomatic or who have multifactorial disease drivers might benefit from adjunctive strategies targeting the deeper underpinnings of prostatic hyperplasia [65].

### 8.2. Anti-Inflammatory Approaches

Growing recognition of inflammation as a key factor in BPH has spurred interest in anti-inflammatory therapies [65,82]. Nonsteroidal anti-inflammatory drugs (NSAIDs) suppress prostaglandin production by inhibiting cyclooxygenases (COX), and observational studies loosely correlate chronic NSAID use with smaller prostate volumes and fewer urinary symptoms [100,101]. However, prospective controlled trials remain limited, and the feasibility of long-term NSAID therapy is constrained by gastrointestinal and renal side effects, especially among older adults with multiple comorbidities [100,101,102].

Selective COX-2 inhibitors provide more targeted anti-inflammatory action, and preliminary investigations suggest that they may impede prostatic hyperplasia and promote apoptosis [76,77,103,104]. Still, cardiovascular safety concerns restrict their prolonged use [103]. Corticosteroids, though potent immunosuppressants, pose substantial risks (e.g., metabolic and infectious complications) that generally exclude them from routine BPH therapy [65].

To limit adverse effects, novel anti-inflammatory agents such as diacerein—commonly used for osteoarthritis—have shown potential in reducing OS and modulating apoptosis in prostatic models [105]. Likewise, curcumin and resveratrol, recognized for their capacity to suppress cytokine secretion and scavenge radicals, display favorable safety profiles, yet large-scale human trials remain inconclusive [76,77].

Phosphodiesterase-5 (PDE-5) inhibitors, initially utilized for erectile dysfunction, also exhibit moderate anti-inflammatory properties and smooth muscle relaxation [94,106]. While this dual role can simultaneously address erectile dysfunction and LUTS, their anti-inflammatory impact may be less direct than that of dedicated anti-inflammatory medications.

### 8.3. Dietary Antioxidants and Nutritional Supplements

Dietary strategies aiming to supply exogenous antioxidants have garnered attention for their ability to neutralize reactive species and augment endogenous antioxidant enzyme capacities [107,108]. Selenium, critical for some antioxidant enzymes, attracted interest following observational findings, but randomized trials—including the SELECT study—failed to confirm protective benefits and even raised concerns about potential links to high-grade cancers in certain subgroups [109,110,111]. Similarly, vitamin E, once advocated for prostate health, has demonstrated inconsistent findings in large interventions, leaving its value in preventing BPH or prostate cancer uncertain [109,110,111].

Among herbal supplements, *Serenoa repens* (saw palmetto) remains popular due to its mild antiandrogenic effects and putative antioxidant or anti-inflammatory actions [112,113,114,115]. Nonetheless, controlled trials have produced conflicting data regarding its capacity to alleviate LUTS or reduce prostate size [115]. The bioavailability and individual metabolism of such compounds can vary significantly, potentially contributing to inconsistent clinical outcomes [107,108,116]. In practice, men frequently combine these supplements with standard therapies, aspiring to synergistic outcomes; however, without robust large-scale evidence, definitive conclusions remain elusive [107,108].

### 8.4. Vitamin D Receptor Agonists

Interest in vitamin D receptor (VDR) agonists has risen due to VDR’s involvement in cell proliferation, immune modulation, and redox homeostasis [75,117]. The binding of calcitriol, the active vitamin D metabolite, to VDR downregulates proinflammatory mediators (e.g., interleukin-8) [75], inhibits COX-2 expression, and enhances antioxidant enzyme production [117]. Collectively, these effects may help mitigate the OS linked to BPH progression [75,117]. Epidemiological data sporadically link adequate serum vitamin D levels to improved prostate health, although large randomized trials remain sparse [117].

Excessive supplementation poses risks of hypercalcemia or hypercalciuria, underscoring the need for clinical caution [117]. Nonetheless, VDR agonists may prove to be beneficial as adjuncts to α-blockers or 5-ARIs by counteracting chronic inflammation and OS [75]. Future research should concentrate on refining dosing regimens, ensuring safety, and identifying the subpopulations most likely to derive therapeutic benefits [117].

### 8.5. Phytotherapeutics

Plant-derived treatments continue to attract interest, notably for individuals with mild LUTS or those intolerant of standard pharmacological options [113,114]. Extracts of *Serenoa repens* and *Pygeum africanum* have longstanding use in various regions, potentially offering mild antiandrogenic effects, local anti-inflammatory action, and antioxidant activity [113,115]. A study evaluating *Curcumae Radix* and *Syzygium aromaticum* extracts in rats indicated synergistic reductions in prostate weight and inflammation [118]. Similarly, *Chaenomeles sinensis* extracts have reportedly inhibited BPH development in rodents by exerting antioxidant and anti-inflammatory effects [119].

Other naturally derived substances—including *Ginkgo biloba* and lycopene-rich tomato derivatives—are under investigation [116,120]. Although some in vitro research has demonstrated anti-inflammatory and antioxidant properties, human trials often yield mixed or modest symptomatic improvements [121]. For lycopene, certain findings associate its antioxidant activity with a reduced incidence of malignancies and possibly slower BPH progression [122]. Nonetheless, extraction methods, dosage variations, and patient selection complicate reproducibility [107]. Even though many patients prefer natural or alternative products, these agents are not devoid of side effects and may interact with established pharmacotherapies [123]. Therefore, well-designed clinical trials that standardize preparations and clarify mechanisms of action are essential for validating their efficacy [108].

**Figure 2:**
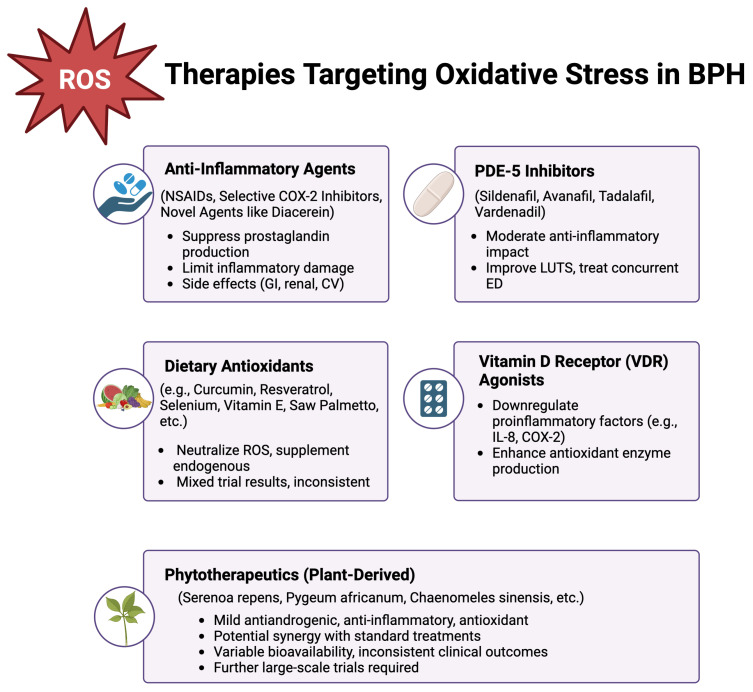
Therapeutic strategies targeting oxidative stress in benign prostatic hyperplasia. *Created in BioRender. Kaltsas, A. (2025)*, https://BioRender.com/m32o053 (accessed on 13 January 2025).

A summary of the current therapeutic strategies, including pharmacological treatments, anti-inflammatory approaches, dietary antioxidants, VDR agonists, and phytotherapeutics, is presented in Figure 2.

## 9. Future Directions

### 9.1. Remaining Gaps in Knowledge

There have been substantial advancements in mapping the complex interplay of OS, inflammation, and the hormonal influences that cause BPH. Nonetheless, essential gaps remain. Investigators have yet to fully determine when subclinical tissue changes progress into symptomatic disease or how to identify early a subgroup of men at risk of rapid progression. Answering these questions could enable timely initiation of BPH treatment before symptoms become overly bothersome. The molecular events linking obesity, metabolic syndrome, and immunologic triggers to the prostatic environment require more in-depth investigation. In addition, large-scale studies providing definitive results on anti-inflammatory and antioxidative agents are still lacking [22,124].

### 9.2. Potential for Novel Biomarkers and Personalized Treatments

An emerging consensus highlights the need for objective markers that can simultaneously capture inflammatory states, ROS levels, and ongoing tissue remodeling. A multi-marker panel of inflammation or OR biomarkers could enable clinicians to identify high-risk patients early, allowing for targeted treatment and making BPH management more individualized. Personalized medicine may rely on identifying genetic polymorphisms in antioxidant enzymes or inflammatory mediators. If certain genetic variants predispose an individual to stronger responses to drugs targeting COX-2 or specific antioxidants, transitioning from standardized drug prescriptions to targeted therapies could significantly optimize outcomes. Advanced imaging modalities, such as multiparametric MRI, could be combined with biomarker results for earlier detection and tailored interventions. Novel combination regimens that address both mechanical and molecular drivers of BPH are also likely to emerge. Researchers may combine α-blockers with antioxidants or anti-inflammatory agents to harness the synergy between relaxing smooth muscle tone and mitigate the oxidative–inflammatory cycles that promote disease progression [125].

## 10. Conclusions

BPH is no longer regarded solely as a hormone-driven overgrowth. A deeper understanding reveals how chronic inflammation, excessive ROS and impaired antioxidant defenses collectively shape the aging prostate. Prolonged OS triggers proliferative signals. This process, when coupled with proinflammatory mediators, exacerbates the imbalance between cell proliferation and apoptosis. The cumulative effect is progressive nodular hyperplasia, often resulting in LUTS that can compromise patient’s quality of life. Although standard treatments such as α-blockers and 5-ARIs address smooth muscle tension and androgenic stimuli, they do not target the underlying oxidative or inflammatory mechanisms. This therapeutic gap creates opportunities to augment or combine these established therapies with interventions that reduce proinflammatory mediator release, suppress ROS formation, or enhance native antioxidant defenses. Examples include NSAIDs, selective COX-2 inhibitors, vitamin D receptor agonists, dietary antioxidants, and herbal extracts. However, each approach is backed with varying levels of supportive evidence and carries distinct risk profiles. Larger prospective studies are needed to determine optimal patient selection, treatment regimens, and dosing strategies for these adjunct therapies. The heterogeneity of BPH suggests that future approaches may shift toward personalized medicine, tailoring interventions to each patient’s oxidative and inflammatory burden, genetic predispositions, and comorbid metabolic factors. A strong rationale supports targeting OS and inflammation as central pathological processes. By mitigating prostatic OS and reshaping the inflammatory microenvironment, men affected by this condition could achieve more stable disease progression, a reduced need for invasive interventions, and an overall improved quality of life.
